# Gene expression in the prefrontal cortex during adolescence: implications for the onset of schizophrenia

**DOI:** 10.1186/1755-8794-2-28

**Published:** 2009-05-20

**Authors:** Laura W Harris, Helen E Lockstone, Phillipp Khaitovich, Cynthia Shannon Weickert, Maree J Webster, Sabine Bahn

**Affiliations:** 1 Institute of Biotechnology, University of Cambridge, Cambridge, UK; 2 Max Planck Institute for Evolutionary Anthropology, Leipzig, Germany; 3 Schizophrenia Research Institute, Prince of Wales Medical Institute, University of New South Wales, Sydney, Australia; 4 Stanley Laboratory of Brain Research, Bethesda, USA

## Abstract

**Background:**

Many critical maturational processes take place in the human brain during postnatal development. In particular, the prefrontal cortex does not reach maturation until late adolescence and this stage is associated with substantial white matter volume increases. Patients with schizophrenia and other major psychiatric disorders tend to first present with overt symptoms during late adolescence/early adulthood and it has been proposed that this developmental stage represents a "window of vulnerability".

**Methods:**

In this study we used whole genome microarrays to measure gene expression in post mortem prefrontal cortex tissue from human individuals ranging in age from 0 to 49 years. To identify genes specifically altered in the late adolescent period, we applied a template matching procedure. Genes were identified which showed a significant correlation to a template showing a peak of expression between ages 15 and 25.

**Results:**

Approximately 2000 genes displayed an expression pattern that was significantly correlated (positively or negatively) with the template. In the majority of cases, these genes in fact reached a plateau during adolescence with only subtle changes thereafter. These include a number of genes previously associated with schizophrenia including the susceptibility gene neuregulin 1 (NRG1). Functional profiling revealed peak expression in late adolescence for genes associated with energy metabolism and protein and lipid synthesis, together with decreases for genes involved in glutamate and neuropeptide signalling and neuronal development/plasticity. Strikingly, eight myelin-related genes previously found decreased in schizophrenia brain tissue showed a peak in their expression levels in late adolescence, while the single myelin gene reported increased in patients with schizophrenia was decreased in late adolescence.

**Conclusion:**

The observed changes imply that molecular mechanisms critical for adolescent brain development are disturbed in schizophrenia patients.

## Background

The human prefrontal cortex is amongst the most phylogenetically recent regions of the brain, and ontogenically, is one of the last to mature [1,2]. The region does not reach adult volume until 10 years of age [3], and myelination continues to progress through adolescence well into early adulthood [4]. A rapid loss of prefrontal grey matter also occurs during adolescence [5,6], which is commonly attributed to an increase in synaptic pruning [7,8]. This peak and subsequent decrease in grey matter volume during late adolescence is a notable feature of the development of the prefrontal cortex and is not observed in other cortical regions [7,2]. The late maturation of this brain region functionally maps to the later development of higher cognitive processes, particularly executive function, social cognition and judgement [9,10]. This period of cognitive development also represents a time of increased vulnerability to the effects of emotional stress, illicit drug-taking, alcohol and nicotine exposure, and is the most common age for patients to present with the symptoms of major psychiatric disorders such as schizophrenia, bipolar disorder and depression [11,12]. Thus characterising the functional alterations occurring in the brains of teenagers and young adults is an important area of study.

To date, the majority of studies of the human adolescent prefrontal cortex have employed brain imaging techniques [2,6,13], with a limited number of histological studies [14,8,15]. Animal studies have provided further evidence for structural remodelling of the prefrontal cortex in adolescence (reviewed in [16,12,17]) but little is known about the molecular mechanisms underlying this process. Several recent post mortem studies of the human prefrontal cortex have begun to address the lack of knowledge in this area by characterising the expression of key genes across post-natal life-span [18-24]. Extending this concept, microarrays can be employed to assess such patterns for thousands of genes simultaneously, an approach which has recently been employed to investigate gene expression patterns in the prefrontal cortex from young adulthood to old age [25].

Using whole genome microarrays we have investigated gene expression in post-mortem prefrontal cortex tissue from healthy individuals aged from birth to middle age. Preliminary analysis of these data focused on gender differences in postnatal development [26]. In the present study, we focus specifically on the period of late adolescence, with the goal of identifying genes whose expression is altered during this period. The data are presented in the context of identifying the molecular processes that are the most likely candidates for the susceptibility to psychiatric dysfunction occurring in late adolescence.

## Methods

### Tissue samples

Fresh frozen *post mortem *prefrontal cortex tissue (Brodman area 46) from 48 individuals varying in age from 0 to 49 years was obtained from the National Child Health and Human Development Brain and Tissue Bank for Developmental Disorders at the University of Maryland, Baltimore, USA (UMBB) (NICHHD Contract number NO1-HD8-3283). The collection protocol was reviewed and approved by the Institutional Review Board of the University of Maryland, Baltimore. The study conforms with the Code of Ethics of The World Medical Association (Declaration of Helsinki).

Subjects were defined as normal controls by forensic pathologists at the UMBB, having no history of psychiatric or neurological complaints, confirmed by next of kin interview. The samples comprised 30 males and 18 females, mean pH was 6.7 ± 0.17 and *post-mortem *interval (PMI) was 18 ± 7.5 hours. Full demographic details can be found in Additional File 1.

### RNA extraction and chip hybridization

All procedures have previously been described [27]. In brief, total RNA was extracted from prefrontal cortex grey matter samples using Trizol (Sigma) and RNA quality was assessed using a high-resolution electrophoresis system (Agilent Technologies) (Additional File 1). Isolated total RNA was then carried through the Affymetrix preparation protocol [28] and each sample was hybridized to one HG-U133 Plus 2.0 GeneChip (Affymetrix) to assess gene expression for the whole human genome.

### Microarray data pre-processing

Raw data were processed and analysed using the R statistical program [29] and Bioconductor packages [30]. Our quality control procedures for Affymetrix microarray data obtained from human post-mortem brain tissue have previously been described [31,27]. Briefly, these included an assessment of chip quality using the AffyPLM package [32] to fit a probe level model to the data, calculation of pairwise correlation coefficients between chips and boxplots of RMA normalised expression values for each chip to identify outlier chips. Based on these analyses, 4 samples were considered outliers and removed from further analysis. Three were from subjects aged less than one year; however, as there were several other samples of similar age, the removal of outliers did not substantially alter the distribution of ages in this series. Normalised expression values (log base 2) for each probe-set on the 44 chips passing our stringent quality standards were computed using the robust multi-chip average (RMA) method [33]. Data were submitted to the GEO archive, with series accession GSE13564.

### Template matching

**Figure 1:**
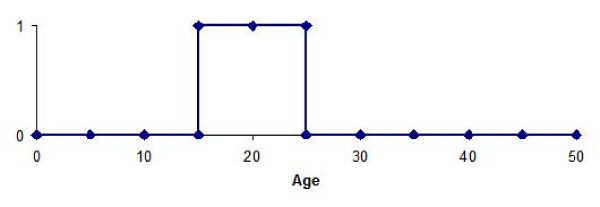
**Template design**. Subjects aged between 15 and 25 years old were assigned a value of one and all other samples a value of zero. The correlation between expression values in the 15–25 group and other samples is tested for each probe-set in turn, using Spearman rank correlation test.

A method of template matching was used to identify probe-sets whose expression profile with age was significantly correlated with a pre-defined template. The age range of interest was defined as 15–25, corresponding to the peak period for onset of schizophrenia [34,35]. The template was a simple step design with samples from subjects aged 15–25 assigned a value of 1 and all other samples assigned a value of 0 (Figure 1). A gene with relatively high expression levels in the 15–25 year age-range compared to other ages would produce a strong positive correlation with the template. Conversely, a gene showing relatively low expression in the 15–25 year age-range would produce a strong negative correlation and thus profiles matching the template or its inverse can be detected simultaneously. Spearman's correlation test was used to identify probe-sets matching the template, using the 'cor.test' function within R [29]. Raw p-values for the correlation tests were extracted and adjusted for multiple testing using the false discovery rate method (FDR) of Benjamini and Hochberg [36]. Adjusted p values are referred to using the notation q throughout and where multiple probe-sets exist for a gene the result for the most significant probe-set is reported.

### Functional profiling

Gene Set Enrichment Analysis (GSEA) [37] was used to identify functionally related groups of genes whose expression pattern was correlated with the template. This algorithm identifies groups of genes which are enriched towards the top or bottom of a ranked list of genes based on a running sum statistic. To rank the genes, Affymetrix probe-set identifiers were converted to HUGO gene symbols using OntoTranslate [38], which were then ranked in order of strength of correlation with the template (most positive to most negative Spearman's correlation coefficient). Where a gene was represented by multiple probe-sets, the probe-set with the strongest correlation was used, leaving a single value for each gene. The pre-ranked list was submitted to GSEA using the default parameters and a list of biological process categories from the Gene Ontology consortium [39] as the gene sets database. Categories with fewer than 15, or greater than 500 members, were excluded from the analysis. The default FDR p value cut-off within GSEA is 0.25, however in the present analysis the significance cut-off was reduced to 0.20 due to the large number of significant categories. This adjustment did not qualitatively alter the results. Next, the leading edge analysis tool within GSEA [37] was used to cluster significant categories for which common genes accounted for the core enrichment signal. This tool clusters gene sets based on the ratio of the intersection and union of genes in the leading edge; a value of more than 0.25 was required for genesets to be considered to cluster together.

### QPCR validation

For quantitative real-time PCR (QPCR), complementary DNA was synthesized from 1 μg total RNA with an oligonucleotide deoxythymidine primer and Superscript First-Strand synthesis system (Invitrogen). QPCR was performed using the Applied Biosystems 7900 HT Sequence Detection System following manufacturer's instructions. Four genes of interest were selected for validation using TaqMan gene expression assays (Applied Biosystems) (glucose metabolism genes PKBFB2 and ACADSB, myelin component MBP, and the schizophrenia risk factor gene NRG1 (probe chosen to be non-isoform specific)). To avoid any potential amplification of genomic DNA, chosen QPCR assays spanned intron-exon boundaries. Additionally, the assays were designed to detect the same or very similar transcript populations measured by the corresponding significant microarray probeset. PPIA, a standard endogenous control from Applied Biosystems was chosen for the normalization of all target genes as it was consistently expressed in microarray samples, and showed no correlation with age or with the template. Triplicate Ct values were generated for all assays and the median value in each case was used for subsequent analysis. Standard curves were constructed for each assay to ensure adequate amplification efficiencies and comparable data across all assays. The relative standard curve method was also employed for quantification of the transcript expression levels.

## Results

Gene expression was examined in a series of post-mortem brain tissue samples obtained from 44 healthy individuals aged from 0 to 50 years of age with the aim of identifying genes whose expression levels were at their highest or lowest levels during late adolescence/early adulthood.

### Correlation of probe-set expression profiles with pre-defined template

For each probe-set, we assessed the correlation between the expression profile with age and a template that described a peak or trough in expression level between ages 15 and 25. A total of 3244 probe-sets were significantly correlated with the template (Spearman's rank correlation test, q < 0.05) after correcting for multiple testing, out of a total of 54675 probe-sets. Of these, 1839 (57%) showed a positive correlation with the template, while 1405 (43%) showed a negative correlation (Additional File 2).

The 3244 significant probe-sets represented 2348 annotated genes (based on Entrez ID information in the Affymetrix HG-U133 Plus 2.0 annotation file, March 2007). Where multiple probe-sets existed for a gene we have reported the result for the most significant probe-set. As expected, consistent results between multiple probe-sets for the same gene were obtained for almost all genes. However, six genes had one probe-set that showed opposite correlation with the template to others for the same gene and these were excluded from further analyses.

Examination of the expression profiles of the most significantly correlated genes revealed that in many cases, rather than showing a precise relationship to the template, gene expression showed a steep change during early development, plateauing during the late adolescent period, with only subtle changes thereafter. To verify that our results reflected changes occurring in the adolescent period, and were not driven by the more dramatic early changes, we tested alternative more complex templates which took the early changes into account; these templates gave highly similar results to the original (data not shown).

### Genes correlated with the adolescence template

**Figure 2:**
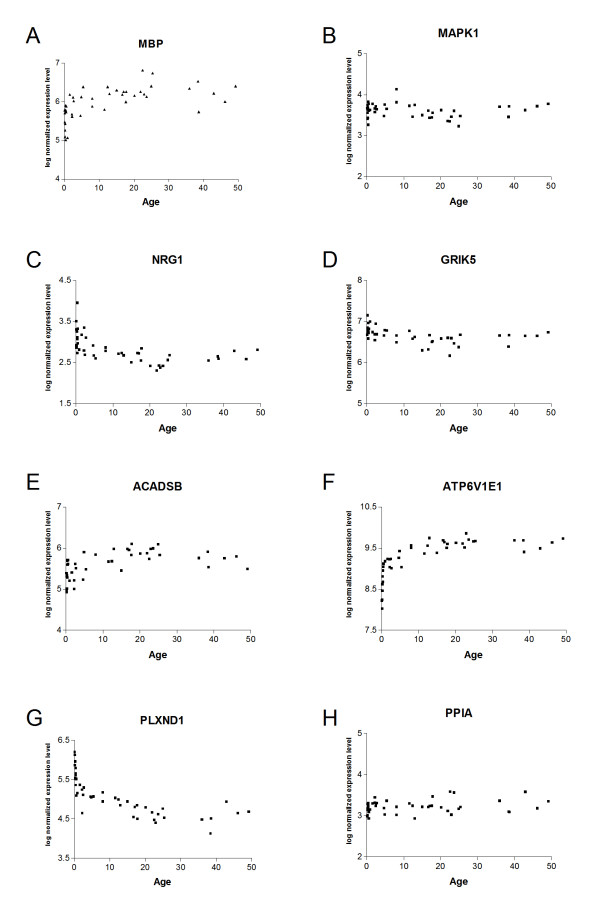
**Expression plots**. Expression levels (log normalized; arbitrary units) versus age (years) were plotted for selected genes of interest which were significantly correlated with a template showing a peak of expression in the age range 15–25: (a) myelin component myelin binding protein (MBP) (r = 0.51, q = 0.020) (b) signaling molecule MAP kinase-1 (MAPK1) (r = -0.55, q = 0.010); (c) glucose metabolism gene acyl coA dehydrogenase (ACADSB) (r = 0.59, q = 0.008) and (d) the electron transport chain component ATP6V1E1 (r = 0.54, q = 0.014); (e) schizophrenia candidate risk factor gene neuregulin-1 (NRG1) (r = -0.59, q = 0.008); (f) ionotropic glutamate receptor subunit GRIK5 (r = -0.60, q = 0.007); (g) axon guidance molecule plexin-D1 (r = -0.56, q = 0.011). For comparison, the housekeeping gene (h) PPIA is also shown (r = 0.14, q = 0.758).

A complete list of genes significantly correlated with the template can be found in Additional File 2 and representative expression plots for several genes of interest are shown in Figure 2. For many of these genes, the most dramatic changes in gene expression occur in early post-natal development (0–2 years), reaching a peak around adolescence and showing only subtle changes in expression thereafter. A number of genes relevant to the field of psychiatric research were identified in this analysis; of note are neuregulin (*NRG1*) and its ligand *ERBB4*, which were both significantly negatively correlated with the 15–25 template (q = 0.008 and 0.011 respectively). *NRG1 *remains one of the more convincing genes to show genetic linkage to schizophrenia in multiple studies [40,41], albeit with a small effect size. NRG1 exists in multiple isoforms, and two independent studies have shown that the type I isoform is selectively increased in schizophrenia [42,43] and the recently described type IV isoform has also been implicated [43]. The HG-U133 Plus 2.0 array contains 5 probe-sets for NRG1, two of which were significant in our analysis; all of these are annotated as targeting the type I isoform and most target multiple isoforms. Interestingly, the most significant probe-set is the only one which targets type IV with 100% coverage; further investigation by microarray or QPCR using isoform-specific probes is required to confirm expression of NRGI isoforms during adolescence. The other major schizophrenia candidate risk factor genes, *COMT *(r = -0.33; q = 0.16), *DTNBP1 *(r = 0.35; q = 0.14), *DISC1 *(r = -0.40; q = 0.08) and *RGS4 *(r = 0.39; q = 0.09), all showed trends but did not reach statistical significance for correlation with the template.

### Functional profiling

**Figure 3:**
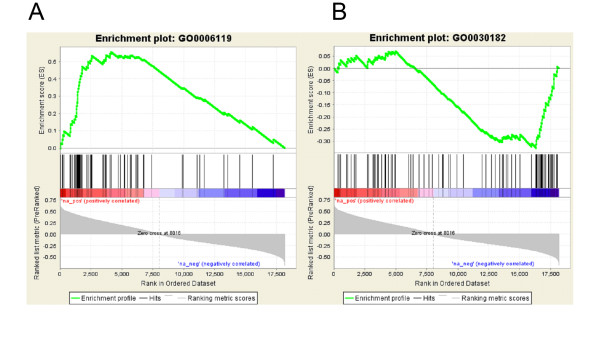
**Functional profiling**. Example enrichment plots are shown for categories identified using GSEA as significantly enriched in either the (a) positively or (b) negatively correlated genes. Black bars represent the position of members of the category in the ranked list, together with the running enrichment score (plotted in green). The leading edge is defined as those genes in the gene set that appear in the ranked list at, or before, the point where the enrichment score reaches its maximum deviation from zero and can be interpreted as the core of a gene set that accounts for the enrichment signal. Examples shown are (a) oxidative phosphorylation (GO:006119) q = 0.000 (b) neuron differentiation (GO:030182) q = 0.143.

GSEA was used to identify functionally defined sets of genes enriched among the genes most strongly positively, or negatively, correlated with the template (Tables 1 and 2, Figure 3). The gene sets investigated in this analysis comprised all biological process categories represented on the HG-U133 Plus 2.0 array as defined by the Gene Ontology (GO) consortium [39] (subject to size filters as described in the Methods section; around 3000 categories in total). As GSEA examines each gene set independently, multiple significant categories containing the same or similar genes can arise due to the nature of the GO hierarchy. We therefore used the leading edge analysis tool within GSEA to identify and group related gene sets, i.e. those in which the significance is driven by an overlapping subset of genes (the "leading edge"). In Tables 1 and 2, the range of q values for grouped gene sets is indicated. Gene sets/groups of gene sets that are functionally associated but are composed of non-overlapping genes in the leading edge are indicated by sub-headings in the tables. Results are presented using the relatively relaxed q-value cut-off of 0.2; however the majority of overlapping gene sets has at least one member with a significance level of q < 0.1. Full details of significant gene sets can be found in Additional File 3.

**Table 1:** Functional profiling, positively correlated genes

**Functional group**	**Gene set name**	**No. of related sets**	**No of members in gene set** **(range)**	**FDR *q *value** **(range)**
Vesicle trafficking	Membrane fusion	2	27–52	0.022–0.026
	Vesicle transport	9	16–348	0.002–0.149
	Vesicle docking during exocytosis	1	21	0.141

Transcription/translation	Ribosome function	4	69–89	0.042–0.179
	mRNA processing	5	157–452	0.056–0.166
	Transcription from RNA pol III promoter	1	25	0.089
	mRNA catabolism	2	17–23	0.027–0.125
	translation	3	30–157	0.012–0.062

Protein transport/metabolism	Protein transport	9	17–294	0.012–0.129
	Ubiquination/proteolysis	11	133–448	0.013–0.024
	Protein folding	1	195	0.003
	Carboxylic acid metabolism	7	17–410	0.087–0.195
	Aspartate family amino acid metabolism	1	18	0.016
	Amine metabolism	2	55–57	0.142–0.152

Electron transport chain	Coenzyme metabolism	4	85–159	0.007–0.029
	Nucleotide metabolism	16	53–178	0.014–0.126
	ATP metabolism	9	36–490	0.012–0.192
	Oxidative phosphorylation	4	21–63	<0.001
	Electron transport	1	289	0.022
	Mitochondrial membrane	2	19–21	0.027–0.149

Glycolysis	Aerobic respiration	7	22–32	0.005–0.016
	Glucose metabolism	13	20–138	0.005–0.079

Oxidative stress	Response to oxidative stress	2	44–63	0.069–0.164
	Peroxisome organization and biogenesis	1	17	0.164

Cell cycle	Mitotic cell cycle	5	17–60	0.077–0.144
	Induction of apoptosis by intracellular signals	1	20	0.067

Lipid metabolism	Sphingomyelin synthesis	3	17–41	0.035–0.187
	Lipid modification	2	15–19	0.032–0.041
	Phospholipid metabolism	6	54–427	0.012–0.054
	Lipoprotein metabolism	3	29–42	0.027–0.069
	Glycerolipid metabolism	6	19–39	0.013–0.180

Others	Iron compound metabolism	6	15–52	0.002–0.053
	Autophagy	1	16	0.062
	Polysaccharide biosynthesis	1	18	0.120
	Biopolymer biosynthesis	1	18	0.128
	Telomere maintenance	1	21	0.132
	Respiratory gaseous exchange	1	17	0.188
	Regulation of Wnt receptor signalling	1	15	0.188
	Vitamin metabolism	1	48	0.188

Functional gene sets are shown which show enrichment in the list of genes positively correlated with a template showing peak expression in the 15–25 age range. Related gene sets as defined by leading edge analysis are grouped together in column 2 and the range of *q *values for these gene sets is indicated. Gene sets/groups of gene sets which are functionally associated but are composed of non-overlapping genes in the leading edge are indicated by headings in column 1. A full detailed list of significant gene sets can be found in Additional File 3.

**Table 2:** Functional profiling, negatively correlated genes

**Functional group**	**Gene set name**	**No. of related sets**	**Gene set size** **(range)**	**FDR q value** **(range)**
Neuron development	Axon guidance	1	26	0.156
	Learning and/or memory	1	15	0.152
	Neuron development	10	47–246	0.074 – 0.165
	Cell development	7	36–425	0.059 – 0.192
	Chemotaxis	1	113	0.194
	Cell motility	4	94–219	0.151 – 0.167
	Cell adhesion	4	37–178	0.066 – 0.177
	Cell recognition	1	16–26	0.158

Neurotransmitter signalling	Neuropeptide signalling pathway	1	72	0.008
	Glutamate signalling pathway	1	18	0.198

Receptor signalling	Enzyme linked receptor protein signaling	2	128–176	0.046 – 0.047
	Regulation of G protein coupled receptor protein	1	25	0.194

Ion transport	Cation transport	2	79–120	0.052 – 0.130
	Regulation of heart contraction	2	22–106	0.153 – 0.158

Protein processing	Positive regulation of protein metabolism	1	51	0.061
	Positive regulation of protein kinase activity	1	56	0.149
	Proteoglycan biosynthesis	1	17	0.181
	Protein polymerization	1	34	0.199

Others	Detection of stimulus	4	19–34	0.116–0.163
	Regulation of cell shape	1	30	0.076

Functional gene sets are shown which show enrichment in the list of genes negatively correlated with a template showing peak expression in the 15–25 age range. Related gene sets as defined by leading edge analysis are grouped together in column 2 and the range of *q *values for these gene sets is indicated. Gene sets/groups of gene sets which are functionally associated but are composed of non-overlapping genes in the leading edge are indicated by headings in column 1. A full detailed list of significant gene sets can be found in Additional File 3.

A large number of the categories significantly enriched among genes positively correlated with the template are associated with energy metabolism, including glycolysis, the tricarboxylic acid cycle, oxidative phosphorylation, the electron transport chain, ATP synthesis and mitochondrial membrane function. Related to these processes are categories pertaining to the oxidative stress response. Other major positively correlated categories include transcription/translational processes and protein trafficking and turnover, suggesting that increased levels of these cellular processes may occur in the cells of the adolescent prefrontal cortex.

The majority of the categories enriched among negatively correlated genes are related to neuronal developmental processes, such as axon guidance, morphogenesis and synaptogenesis. Genes related to glutamate signalling, including *GRM4*, *GRIK5 *and *GRIN3A*, are decreased, as are neuropeptide signalling genes. No genes or categories relating to dopamine or 5 HT signalling were found significantly altered in the current analysis. Due to the filtering parameters for category size, categories relating to GABA signalling were excluded from the analysis; however two GABA receptor subunits were significantly correlated with the template (*GABRG1*, -0.46, q = 0.037; *GABRG2*, r = 0.45 q = 0.041).

As additional validation of the functional profiling, a repeat analysis was carried out using a different approach implemented in the software GoSTAT [44]. This algorithm examines a list of significant genes and identifies overrepresentation of GO categories in the list, relative to their representation on the entire chip. This analysis gave qualitatively very similar results to those obtained using GSEA (data not shown).

### Myelination

Due to the strong evidence for white matter alterations during adolescent brain development, and evidence for the involvement of aberrant myelination in major neuropsychiatric disorders, we predicted that genes related to myelination would be detected in this analysis. Several GO categories relating to myelin sheath synthesis and membrane lipid metabolism were significant (q < 0.2, Table 1). Other categories containing the keyword 'myelin' did not pass the minimum size filter (i.e. contained fewer than 15 genes) and therefore could not be tested.

To further investigate myelination processes in this dataset, we turned to individual genes. We previously identified differential expression of key genes associated with the mature myelinating oligodendrocyte in the brains of schizophrenia patients [45], many of which have since been replicated in independent studies,[46-51]. More recently, we have shown upregulation of the *ASPA *enzyme, important in white matter maintenance, in schizophrenia [52]. We assessed the present dataset for 11 specific myelination genes previously reported dysregulated in schizophrenia brain tissue. Of the 10 genes previously shown to be downregulated in the brains of schizophrenic patients, 8 were significantly positively correlated with the template, i.e. they reached a peak in their expression levels during late adolescence (Table 3). The other two genes were also positively correlated but did not quite reach significance (*CLDN11 *r = 0.42, q = 0.06; *MOBP *r = 0.35, q = 0.14). Strikingly, the one gene that had previously been found upregulated in schizophrenia, *MPZL1*, was significantly negatively correlated with the template i.e. expressed at relatively low levels during adolescence (Table 3).

**Table 3:** Myelination related genes showing expression changes during adolescence

**Gene**	**Symbol**	**Correlation with 15–25 template**		**Change in SZ**	**Number of studies**
		***r***	***Adjusted p-value***		
				
Claudin 11	CLDN11	0.42	0.0613	Down	3 ^(2,4,6)^
2',3'-cyclic nucleotide 3' phosphodiesterase	CNP	0.49	0.0258	Down	4 ^(2,3,4)^
myelin associated glycoprotein	MAG	0.44	0.0496	Down	4 ^(1,2,3,4)^
mal, T-cell differentiation protein	MAL	0.44	0.0471	Down	3 ^(1,3,5)^
myelin basic protein	MBP	0.51	0.0201	Down	2 ^(5,6)^
Myelin-associated oligodendrocytic basic protein	MOBP	0.35	0.1384	Down	2 ^(5,6)^
myelin oligodendrocyte glycoprotein	MOG	0.49	0.0271	Down	2 ^(4,6)^
plasma membrane proteolipid (plasmolipin)	PLLP	0.48	0.0285	Down	1 ^(1)^
proteolipid protein 1	PLP1	0.46	0.0386	Down	2 ^(1,6)^
myelin protein zero-like 1	MPZL1	-0.63	0.0063	Up	1 ^(6)^
aspartoacylase	ASPA	0.48	0.0298	Down	1 ^(7)^

Myelination related genes showing expression changes during adolescence and relevant findings from the schizophrenia literature are shown. Citations are indicated by a footnote in the final column as follows: 1 [46] 2. [47] 3. [48] 4. [49] 5[50] 6. [45] 7. [52]

### Validation

**Figure 4:**
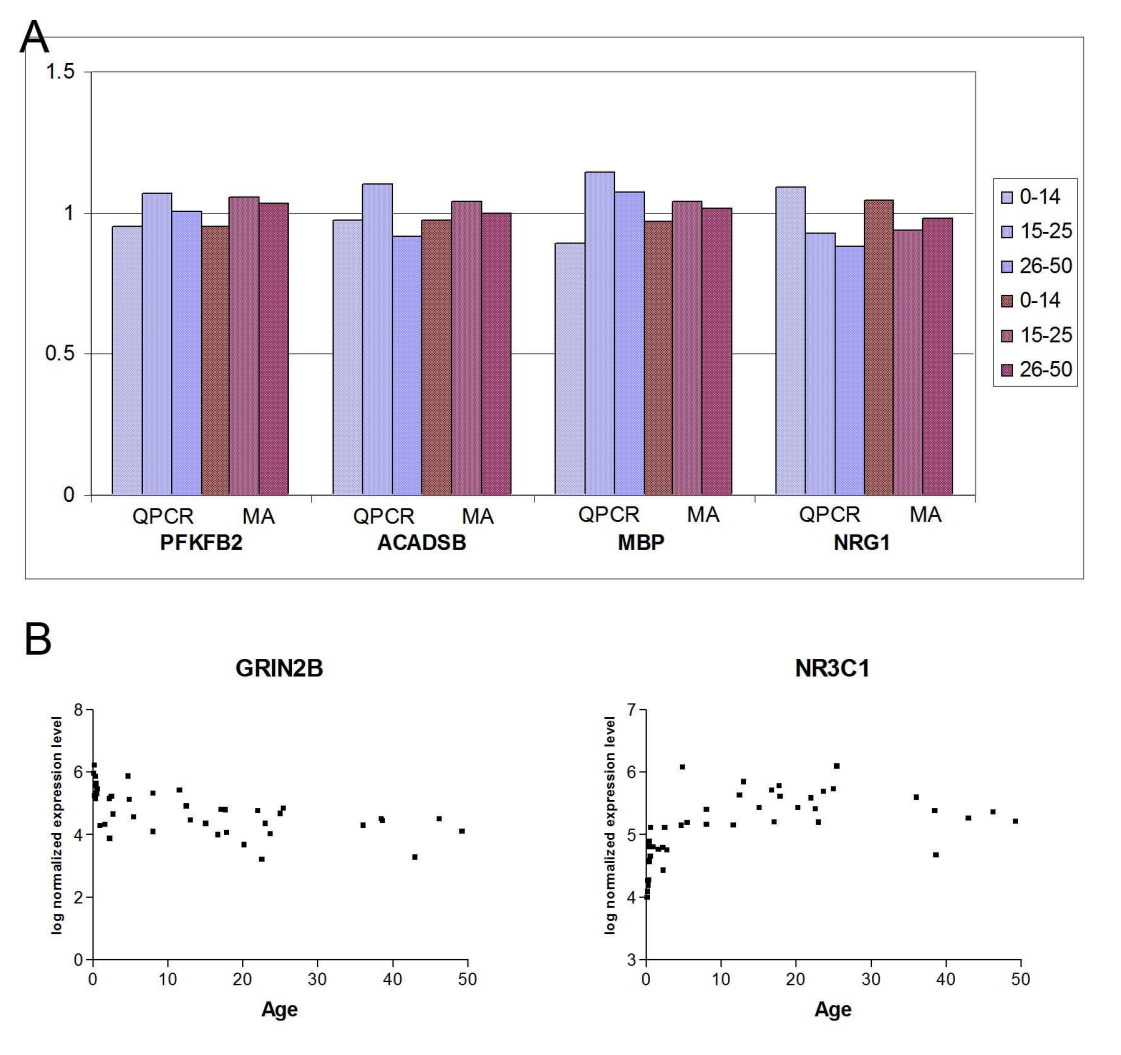
**Validation**. (a) Quantitative real-time PCR (QPCR) validation of microarray (MA) data for 4 genes, PFKFB2, NRG1, ACADSB and MBP. Expression values shown are fold change in each of 3 age groups relative to the mean for the whole dataset. (b) Plots showing log normalized expression levels (arbitrary units) versus age (years) of two genes, NMDA receptor subunit 2B (GRIN2B) and the glucocorticoid receptor NR3C1, which show the expected expression pattern based on previous studies of the developing primate prefrontal cortex.

Quantitiative real time PCR validation of selected gene expression patterns was performed. Although the pattern of developmental expression changes in the microarray data was clear, the differences between individual samples were in most cases small, and we were unable to replicate the template matching results using QPCR due to the increased amount of variability in this dataset. This is likely to have arisen from the high potential for small variations in the reaction setup with this method and the problems associated with normalising to a housekeeping gene [53]. However, by examining the fold change in expression level in the three age groups (0–14, 15–25, 26–50) relative to the mean of all 44 samples, comparable results from both microarray and QPCR data are revealed (Figure 4). It should be noted, however, that the QPCR data suggest *NRG1 *expression levels continue to decrease after adolescence, whereas the microarray data indicates a plateau effect.

To further demonstrate the validity of the results, we took a second approach of using the microarray data to show previously reported developmental alterations in gene expression. Literature searches were undertaken to identify genes that have been reliably shown to be altered in the developing primate prefrontal cortex. The NMDA receptor subunit *NR2B *(*GRIN2B*) decreases postnatally to a constant level in both rodent and primate prefrontal cortex [54,55] and this pattern is reflected in our data (Figure 4a). Secondly, the glucocorticoid receptor gene is one of the few that has previously been demonstrated to show a specific change during adolescence in the human prefrontal cortex (in an independent brain series) [19]. This finding was replicated in our results (Figure 4b, with the glucocorticoid receptor gene *NR3C1 *showing a highly significant positive correlation with the template (q = 0.008) and all 5 probe-sets for this gene showed q values ≤ 0.07.

### Demographics

The demographic variables brain pH and PMI (see Methods and Additional File 1) were assessed for correlation with the template using Spearman's test and neither were significant (pH: r = 0.08, q = 0.60; PMI: r = -0.29, q = 0.11). The categorical variable gender was also assessed using Fisher's exact test to compare the distribution for the 12 samples in the 15–25 age range (7 male, 5 female) and the other 32 samples (20 male, 12 female); no significant difference was found (Fisher's p = 1.00). These results indicate that pH, PMI and gender did not introduce a bias among the samples that could account for the changes in gene expression observed in late adolescence.

## Discussion

In the present study, we have profiled gene expression during normal postnatal development of the human prefrontal cortex, spanning the period from birth to middle age. We designed a template to identify genes whose expression is altered during late adolescence/early adulthood (defined as ages 15–25), compared to younger and older ages. Many genes detected showed dramatic changes in early development to reach a plateau in adolescence with relatively subtle changes thereafter. One caveat to these data is the lack of any samples from individuals aged between 26 and 35; thus the expression levels of identified genes between these ages are unknown. However, it would seem reasonable to assume that they would lie on a curve fitted to the known datapoints.

We have functionally profiled the genes to provide insight into the biological processes occurring as the prefrontal cortex matures. Although data gained from functional profiling studies are some way from providing hard cellular evidence that these biological processes are altered, they can provide a useful indication of the most likely candidates, given the inherent problems of studying human brain function at the molecular level.

The majority of gene categories showing relatively low expression levels during adolescence are related to neuronal developmental process, such as axon guidance, morphogenesis and synaptogenesis. The most significantly altered genes in these categories include growth cone guidance molecules (eg netrins, semaphorins and the semaphorin receptor neuropilin), and also neurexin and neurolignin which link the pre-and post-synaptic machinery and may control the balance of excitatory and inhibitory synapses [56]. The expression profile of these genes, especially those involved in synaptic maintenance such as neurexin, suggests axon retraction and is consistent with increased synaptic pruning in this developmental period [7,8]. In recent years it has been debated whether the decrease in grey matter volume in the adolescent prefrontal cortex found in brain imaging studies is a true reflection of synaptic loss or merely an artefactual representation of increased white matter volume [2,6]. Our data provide evidence at the gene expression level that there are indeed alterations in processes associated with synaptic development during adolescence, in addition to increased expression of myelination genes. As all samples were dissected to contain similar amounts of grey (majority) and white matter (trace), it is unlikely that the results could be a simple reflection of increased white matter density in the adolescent samples.

The pattern of changes detailed in this study indicates that genes associated with energy generation via glycolysis and oxidative phosphorylation reach peak expression during adolescence, coupled with other active cellular processes such as transcription, translation and protein transport. This may represent an increase in energy supply to the prefrontal cortex. PET studies have indicated a peak in resting cortical glucose utilisation in childhood with a gradual decline to reach adult values in late adolescence [57]. However more recently, fMRI studies have shown increased activation of the prefrontal cortex in adolescents in certain tasks [58].

It is not clear what function this increased cellular energy supply might support. Based on the evidence for synaptic pruning in this study and others [7,2,8], and a decrease or no change in the expression of neurotransmitter signalling genes, increased neuronal activity seems unlikely to be the explanation (although it cannot be ruled out). Both the current analysis and previous studies suggest that myelination may be the major energy-demanding process occurring in the adolescent prefrontal cortex. Myelin synthesis is an ATP-dependent process [59] and oligodendrocytes normally oxidise glucose and lactate at far higher rates than either neurons or astrocytes, both for energy and directly in lipogenesis [60]. Alterations in many of the other positively correlated functional categories identified here are consistent with increased myelination, including the metabolism, sorting and transport of proteins and lipids [61-64]. Moreover SNARE complex and other related genes are expressed in oligodendrocytes and may be implicated in myelin targeting to the plasma membrane [62,65]; thus a peak in the expression of these genes in adolescence is consistent with increased myelination and may represent an alternative interpretation for their function in adolescence, besides their well-documented role in synaptic vesicle trafficking. Furthermore, although the molecular processes underlying synaptic pruning are not fully understood, it is conceivable that large scale removal of synapses may itself be a drain on energy resources. Indeed, the concurrent strengthening of remaining synapses may well consume ATP [66].

It remains unclear what the control mechanisms for adolescent brain alterations are, although previous studies have pointed towards a complex interaction between hormonal and neural systems. Candidates for the control of this process include the glucocorticoid receptor which shows a profound alteration in expression during adolescence, demonstrated in this and an independent study using a different brain series [19]. Other candidates include the POU factor genes, which have been proposed as master regulators of puberty in the hypothalamus [67-69] and are also involved in the control of myelination processes [70]. The present study suggests that *POU3F2 *expression dips during adolescence in the prefrontal cortex (q = 0.025) and may provide a link between the hormonal control of puberty and the molecular alterations seen in this developmental period.

### Links with schizophrenia

The period of late adolescent development is of particular interest to psychiatry research, as this time window corresponds to the age of onset of major neuropsychiatric disorders, especially schizophrenia. A striking feature of the data presented here is the similarity between genes and processes altered during late adolescence and those known to be dysfunctional in the schizophrenia brain. For example, the leading candidate risk factor gene, NRG1 [40,41], which has also been linked to bipolar disorder [71], is minimally expressed during late adolescence together with its ligand ERBB4. This result is supported by data from [25], showing that the expression of NRG1 and ERBB3 decrease in early adulthood and increase thereafter. Despite a number of recent publications into the molecular function of this gene in schizophrenia [72,73,43], strong evidence for an etiological role in the disorder is lacking. While previous hypotheses have focused on the role of neuregulin in early development as a predisposing factor in schizophrenia, the present data suggest that it has an important additional function in the maturation of the prefrontal cortex and may be one of many factors involved in the "unmasking" of vulnerable processes at this time point.

Neurotransmitter systems that show altered function during adolescence may also be particularly vulnerable to perturbation during this period; our results suggest that neuropeptide and glutamate signalling may be particularly important. There is strong evidence for glutamatergic abnormalities in schizophrenia, not least due to the psychosis-inducing effects of glutamate antagonists such as PCP. Various studies have demonstrated alterations in neuropeptides in neuropsychiatric disorders [74-76]; the alteration in expression of these genes during this critical developmental period, in a region of the brain strongly associated with schizophrenia symptoms, strengthens the evidence for their role in the etiology of schizophrenia. It is of note that among the many other neurotransmitter systems implicated in schizophrenia, only GABA-related genes showed any alterations during late adolescence. Notably, no significant changes were observed in gene expression associated with the dopamine system, suggesting that the reported changes during adolescence [21] may be less pronounced than those in the glutamate and GABA systems.

The present finding of a peak in energy metabolism-related gene expression in the adolescent brain is of particular interest in light of an increasing body of evidence from our laboratory [77-80] and others [81-84] implicating energy metabolism deficits in schizophrenia and, to a lesser extent, bipolar disorder [82,85]. Such alterations have been detected in both the brain and periphery, and are present in first- onset drug-naïve patients. Based on these findings, we hypothesize that individuals who are predisposed to develop schizophrenia are unable to meet the energy demand in critical brain regions during adolescence, precipitating behavioural and cognitive symptoms. At the molecular level the downstream effects of such a deficit are currently unknown. Our finding that myelination genes in schizophrenia are regulated in direct opposition to their expression pattern during adolescent development is consistent with hypotheses of delayed maturation of the prefrontal cortex resulting in decreased white matter volume [86]. More research is clearly needed, but based on current knowledge we propose that disturbances in energy metabolism which may be critical in the adolescent prefrontal cortex have downstream effects on myelination and other developmentally regulated processes such as synaptic plasticity and neurotransmitter function. These processes, or their outcome, have in turn been demonstrated to be abnormal in the brains of schizophrenia patients and are likely candidates for the direct cause of behavioural and cognitive symptoms in the disorder. Furthermore, as *NRG1*/*ERBB4 *function affects many of the above functions, including glutamatergic and GABAergic synapse stabilisation and oligodendrocyte function [87-89], the modulation of *NRG1 *expression during adolescence may represent a point of critical interaction between this genetic risk factor and other abnormal processes in the brain of susceptible individuals.

One limitation of these data lies in the cross-sectional study design, which relies on the assumption that gene expression is comparable in all individuals. In addition, the number of samples in each age range is fairly small and precludes investigation of gene expression patterns in adolescence by gender, for example, which would be of interest. These and other limitations inherent to post mortem studies of the human brain, such as variation in brain pH and PMI between individuals, mean these results must be considered in the context of data from human brain imaging and animal studies, and dynamic studies of human peripheral tissues.

## Conclusion

In summary, our data provide molecular correlates of known functional processes occurring in the developing human prefrontal cortex at the gene expression level. These alterations are highly specific to the period of late adolescence and may represent the molecular foundations of the vulnerability to neuropsychiatric disease which occurs during this developmental time window. Moreover, the processes show an intriguing link with known alterations in the schizophrenia brain, and at the individual gene level, especially in genes related to myelination, we have demonstrated a direct correspondence. Further work is now required on this important brain series to quantify and characterise molecular changes at the protein and functional level.

## Abbreviations

FDR: false discovery rate; GO: Gene Ontology; GSEA: gene set enrichment analysis; PMI: *post mortem *interval; RMA: robust multi-chip average; UMBB: University of Maryland Brain Bank.

## Competing interests

The authors declare that they have no competing interests.

## Authors' contributions

All authors read and approved the manuscript. LH and HL carried out data analysis and interpretation and wrote the manuscript. PK was responsible for microarray hybridization and contributed to the data analysis. CSW and MW conceived the study, were responsible for sample collection, and critically revised the manuscript. SB participated in study design and coordination and critically revised the manuscript.

## Pre-publication history

The pre-publication history for this paper can be accessed here:



## Supplementary Material

Additional file 1 **Full demographic variables and RNA quality rating**. Full demographic details (age, PMI, gender, race, tissue pH) plus RNA integrity number (RIN) are provided for all samples included in the final analysis (i.e. after outlier removal). Click here for file

Additional file 2 **Full list of significant probesets**. Correlation coefficients, significance levels and gene information are provided for all probesets significantly correlated with a template showing a peak of expression between ages 15 and 25 (see text for details). Click here for file

Additional file 3 **Functional profiling results**. The data provided represent the full results of the GSEA functional profiling analysis. The data presented in Tables 1 and 2 of the main manuscript summarise these findings. As GSEA examines each gene set independently, multiple significant categories containing the same or similar genes can arise due to the nature of the GO hierarchy. Main headings indicate groups of functionally related categories. Categories listed under a lower case heading form a cluster as defined by Leading Edge analysis (ie containing overlapping gene lists). Clusters marked with * or # within a main heading show weak similarity to each other. Click here for file
